# Artificial insemination with liquid-stored and fresh semen in grey partridge (*Perdix perdix* L.)

**DOI:** 10.1016/j.psj.2026.107291

**Published:** 2026-06-12

**Authors:** Marcel Bawej, Marta Michalak, Joanna Rosenberger, Artur Kowalczyk

**Affiliations:** Division of Poultry Breeding, Institute of Animal Husbandry and Breeding, Wrocław University of Environmental and Life Sciences, Chełmońskiego 38C, 51-630, Wrocław, Poland

**Keywords:** Artificial insemination, Short-term semen preservation, Semen extender, Sperm dose, Progressive motility

## Abstract

European populations of the grey partridge (*Perdix perdix* L.) have declined rapidly, necessitating captive breeding programs to supplement wild populations. Artificial insemination could optimize these reproductive efforts, yet specific protocols are lacking. This study evaluated the effects of short-term liquid storage (24 h at 5 °C in EK and Lake extenders) on selected semen quality parameters and determined how extender type and sperm dose affects fertility level.

Semen was collected from 15 males, pooled, diluted 1:2 and evaluated in vitro at 0 h and after 24 h of storage. Sperm motility and kinematics were assessed using Sperm Class Analysis system, whereas viability and morphology were evaluated by nigrosin-eosin staining. Obtained data was analyzed using repeated measures ANOVA. Fertilization outcomes, assessed by incubating eggs laid following artificial insemination, were analyzed using generalized linear mixed models.

Storage significantly reduced sperm motility, viability and the proportion of morphologically normal spermatozoa (P < 0.05). The EK extender better preserved sperm viability and normal morphology, whereas the Lake extender maintained a more stable kinetic profile over the time. Fresh ejaculates naturally exhibited a remarkably low proportion of morphologically normal spermatozoa (14.2%). In vivo, sperm dose significantly affected fertilization success (P < 0.05); with higher doses (≥ 16 × 10^6^ spermatozoa) consistently resulting in higher fertility rates in both fresh and stored semen. Extender type did not significantly affect fertilization rates. Progressive motility showed a tendency to predict fertilization success after storage (P = 0.06).

Despite reduced in vitro semen quality, short-term liquid storage of grey partridge semen can be effectively applied in artificial insemination procedures when an adequate sperm dose is used. These findings provide a practical basis for improving reproductive management and developing genetic resource conservation strategies in this species.

## Introduction

Natural populations of grey partridge (*Perdix perdix* L.) in Europe have declined rapidly in recent decades, largely due to habitat degradation, agricultural intensification and increased predator pressure (Laux et al., 2024). Conservation efforts of the grey partridge focus on habitat management, aiming to improve environmental conditions or supplement wild populations with individuals bred in captive breeding programs (Panek, 2005; Bro et al., 2012; Černý et al., 2020; Buckley et al., 2021; Palacios et al., 2024).

Captive breeding relies on natural mating in aviary system, which often leads to behavioral incompatibility, partner aggression and suboptimal reproductive outcomes (Prieto et al., 2018; Delaitre et al., 2023). Although artificial insemination (**AI**) is well established in poultry species and has been successfully applied in several wild gallinaceous species (Kowalczyk et al., 2011; Castillo et al., 2021; Santiago-Moreno and Blesbois, 2022; O’Brien et al., 2023), there is currently a lack of data on the use of AI in the grey partridge (*Perdix perdix*). In particular, the effectiveness of liquid semen storage and its subsequent use for AI in this species has not been evaluated in this species. The development of such protocols is especially challenging in *P. perdix* due to its strictly monogamous mating system and the specific characteristics of its semen, including relatively low sperm concentration and distinct morphological features. These traits may limit semen resilience to handling and short-term storage, making the direct transfer of protocols developed for polygamous gallinaceous birds not effective (Møller, 1988; Møller and Ninni, 1998; Bawej et al., 2026).

Liquid semen storage enables the transport of genetic material between breeding centers without the risks associated with moving live birds and represents an essential step toward the development of cryopreservation and genetic reserve strategies (Ciereszko et al., 2011). However, freshly collected semen rapidly loses viability during in vitro storage, making the duration of liquid storage a critical factor for the effectiveness of AI. Furthermore, successful AI requires an adequate insemination dose of motile spermatozoa to effectively colonize the female’s sperm storage tubules (**SST**) (Blesbois and Brillard, 2007; Abouelezz et al., 2015; Di Iorio et al., 2020; Bustani and Baiee, 2021).

Previous studies in poultry species have shown that a 24-hour storage period is a biologically relevant time frame for evaluating the protective capacity of semen extenders, as it reflects both early semen deterioration and the logistical requirements of semen handling and transport (Kowalczyk et al., 2011; Łukaszewicz et al., 2020; Getachew et al., 2023). Therefore, the aim of this study was to evaluate the effect of short-term semen storage (in EK and Lake extenders) on selected semen quality parameters in the grey partridge, and to determine how extender type and sperm dose affect AI success.

## Materials and methods

### Experimental birds

The study was conducted over two consecutive reproductive seasons using two separate cohorts of grey partridge (15 adult male and 10 female grey partridges in each season). All birds originated from the same source, were hatched during the same period, and were yearlings (one year old) at the onset of the experiments. This age corresponds to sexual maturity, as grey partridges start breeding in the year after hatching (Potts and Aebischer, 1990). Prior to the experimental period, during the autumn and winter months, the birds were housed together in a large outdoor aviary to facilitate natural social interactions. Before the start of the reproductive season (early March) partridges were transferred into individual enclosures (80 × 40 × 50 cm), to prevent aggression resulting from the species' strong territorial instinct during the breeding period. Throughout pre-experimental and experimental stages, the birds were maintained under natural photo period and temperature conditions, with food and water provided *ad libitum.* While housed individually, males and females maintained continuous visual and vocal contact.

### Experimental design

In the first year, fresh semen samples collected from 13 males were evaluated individually and used for AI of 8 females. This reproductive season focused on establishing baseline reproductive performance and verifying the feasibility of AI procedures in *Perdix perdix.* Additionally, to provide a biological baseline for the AI outcomes, natural reproductive behavior was observed in two pairs of grey partridges housed together in joined enclosures. The mean daily copulation frequency was recorded, and the eggs collected from these pairs were artificially incubated to determine the fertilization rate.

The second reproductive season (second year), focused on the efficacy of short-term liquid storage. Semen was collected from 15 males, pooled and subjected to liquid storage in two semen extenders prior to further analyses and AI of 10 females.

This two-stage experimental design was implemented due to biological and ethical constraints associated with small volumes of ejaculates and working with a limited number of birds. Previous studies have shown that the average ejaculate volume in *P. perdix* is approximately 4.4 µL (Bawej et al., 2026), which restricts the number of parallel experimental treatments that can be applied to individual samples. In accordance with the principles of the 3Rs (Replacement, Reduction, and Refinement), pooling ejaculates in the second year allowed sufficient sample volume for extender comparison, semen storage, and in vivo fertilization assessment. Crucially, environmental factors, housing conditions and nutritional regimens were kept identical across both reproductive seasons to minimize potential year effects.

### Sperm collection

Sperm was collected twice or three times a week using the dorsal-abdominal massage adapted to this species (Burrows and Quinn, 1937). Samples were obtained by capillary method using a sterile urological catheter (∅ 1,98 mm) and transferred to Eppendorf tubes and immediately diluted with the respective extender to prevent dehydration and sample loss.

### Liquid semen storage

In second season ejaculates collected from the 15 males were combined into smaller sub-groups to create independent semen pools. Over the experimental period a total of 27 independent pools were generated, of these, 24 pools of sufficient volume were used for AI. Each pool was divided into two parts: semen diluted 1:2 ratio (semen:extender) as suggested by Blesbois and Brillard (2007), with 2 extenders: EK (Łukaszewicz et al., 2020) and Lake (Lake and Ravie, 1984) (samples marked as EK and Lake respectively). Immediately after dilution at room temperature (18 to 20 °C) and evaluation, the Eppendorf tubes were placed directly into a refrigerator set to 5 °C, allowing the samples to passively equilibrate to the target storage temperature. To determine the effect of semen extender on the viability of semen, sperm morphology was evaluated for fresh semen (samples marked as 0 h) and semen after 24 h of storage in temperature 5 °C (samples marked as 24 h), using the nigrosin-eosin staining technique (Łukaszewicz et al., 2008).

### Assessment of sperm quality

The volume of semen and semen doses was defined using an automatic pipette. Sperm concentration was determined by hemocytometer count with the use of eosin-3% NaCl solution and Thoma-Zeiss chamber following the dilution of semen at a 1:200 ratio (1 µL of semen to 200 µL of eosin-3% NaCl solution).

Sperm motility was analyzed using Sperm Class Analyzer (Microptic S.L., Barcelona, Spain). Sperm samples were diluted 1:20 in extender and loaded onto a warmed (38 °C) 20-µm Leja 8-chamber slide (Leja Products B.V., Nieuw-Vennep, The Netherlands). The CASA settings established for the grey partridge were performed at a frame rate at 50 Hz, capturing 50 frames per field. Particle area identification was set between 2 and 20 µm², with a connectivity of 12, spermatozoa with VAP < 5 µm/s were considered immotile. For each sample, a minimum of 500 spermatozoa or at least five microscopic fields were analyzed. The percentage of motile spermatozoa (%**MOT**), and the percentage of spermatozoa showing progressive motility (%**PMOT**) were recorded. Sperm movement characteristics – curvilinear velocity (**VCL**), straight-line velocity (**VSL**), average path velocity (**VAP**), amplitude of lateral head displacement (**ALH**) and linearity (**LIN**) were also analyzed by Sperm Class Analyzer.

The percentage of live and dead spermatozoa and morphological forms of live spermatozoa were examined on nigrosin-eosin smears. Nigrosin-eosin stain was prepared according to Jaśkowski (Głód, 1976). To prepare the smears, 0.2 µL of semen was added directly to 15 µL of the stain using a micropipette. The smears were made directly on a heating plate set to 37 °C to ensure immediate drying. The results of morphological evaluation were expressed as the percentage of eight categories of spermatozoa forms (200 cells = 100%).

### AI and fertility assessment

In the first reproductive season, fresh semen was used for AI of eight females immediately after semen evaluation. A total of 23 inseminations were performed during this season, all utilizing the EK diluent. The maximum time between semen collection and AI did not exceed 20 min. Each female received the entire volume of ejaculate collected from an individual male excluding an aliquot required for the laboratory evaluation.

In the second reproductive season, pooled liquid-stored semen was used for AI of ten females. During this season, a total of 24 inseminations were performed, consisting of 11 inseminations with the EK extender and 13 inseminations with the Lake extender.

Because this study represents the first attempt at artificial insemination in the grey partridge, a biologically established optimal sperm dose for this species is currently unknown. To evaluate the effect of the sperm count on fertilization success, the AI doses from both seasons were categorized into two ranges using an exploratory cut-off: fewer than 16 × 10⁶ live spermatozoa (A) and comprising 16 × 10⁶ or more live spermatozoa (B). This threshold was adopted based on AI protocols described for the red-legged partridge (*Alectoris rufa*) (Abouelezz et al., 2015), which served as the most appropriate proxy species due to its comparable body size and similar monogamous mating system.

In both seasons, AI attempts were performed 2 to 3 times per week at the flock level, corresponding to the frequency of semen collection. Due to the limited volume of ejaculates in the second season, a maximum of two females were inseminated per session. Furthermore, because of the variable individual egg-laying patterns of this wild species, the number of inseminations per female varied from one to four over the reproductive season (totaling 24 AI events for 10 females). Females were re-inseminated only after they had begun laying unfertilized eggs, ensuring the depletion of sperm from the previous AI, which allowed all collected eggs to be reliably attributed to a specific AI event. AI was performed by gently everting the vaginal orifice of the female through light abdominal pressure. The first AI of each female was conducted after the laying of the first egg. Birds were checked daily; laid eggs were collected and stored for a maximum of five days at 15 °C. Eggs were then incubated at 37.8 °C and 55% relative humidity. Fertility was assessed per AI event and for each extender, among eggs laid following a given AI, based on the proportion of eggs showing embryonic development on the 4th day of incubation.

### Statistical analysis

Statistical analyses were performed using Statistica software (version 13.3, TIBCO Software Inc., Palo Alto, CA, USA) and R software (R Core Team, Vienna, Austria). Prior to the main analysis, the normality of the residuals was assessed via the Shapiro-Wilk test.

For the sperm quality assessment, the experimental unit was defined as the pooled ejaculate. To determine the effects of the experimental factors on sperm parameters (viability, kinematics, and morphology), a two-way repeated measures analysis of variance (**RM-ANOVA**) was applied. The statistical model included two within-subject factors: the type of semen extender (two levels: EK and Lake) and the liquid storage time (two levels: 0 h (fresh) and 24 h), as well as their interaction (Extender × Time). When the RM-ANOVA revealed a significant main effect or a significant Extender × Time interaction, differences between specific experimental groups were further analyzed using the Bonferroni post-hoc test for multiple comparisons.

For the in vivo fertilization Generalized Linear Mixed Models (**GLMM**) were applied, where each egg was treated as an individual binary observation (fertilized vs. unfertilized). For the first reproductive season, AI Dose (A vs. B) was included as a fixed categorical effect. In the second reproductive season, both Extender type (EK vs. Lake) and AI Dose (A vs. B) were evaluated as fixed effects. To control for the inherent variation in initial sperm quality, %PMOT was incorporated into both models as a continuous covariate. To account for the repeated measures design and individual physiological variation, Male ID (or Semen Pool ID in second season) and Female ID were fitted as crossed random intercepts. The significance of fixed effects was assessed using Type III Wald chi-square tests. Data was presented as means ± standard error (SE). For all analyses differences were considered statistically significant at P < 0.05 (α = 0.05).

### Ethics approval

The study was conducted according to Resolution No. 021/2023/P1 of 21 June 2023 issued by the Local Ethical Committee for Animal Experiments in Wrocław. Based on the activities described in the application, the Committee discontinued the proceedings, as the research involved routine veterinary procedures and standard agricultural practices related to animal husbandry. The birds were housed in facilities and enclosures designed to meet their behavioral needs.

## Results

### Sperm Motility and Viability

RM-ANOVA showed a highly significant effect of storage on %MOT (P < 0.001) (Table 1.). The percentage of motile spermatozoa decreased after 24 h of liquid storage. However, neither the type of extender (P = 0.184) nor the Extender × Time interaction (P = 0.169) affected this parameter. This indicates that the decline in %MOT was observed in both the EK and Lake extenders.

**Table 1:** Effect of extender type (EK vs. Lake) and storage (fresh semen vs. liquid stored semen) on the motility, kinematics, viability, and morphology of grey partridge (*Perdix perdix*) semen.

Parameter	Fresh semen (0 h)		Liquid stored semen (24 h)		SEM^1^	P-value (Extender)	P-value (Time)	P-value (Interaction)
	EK	Lake	EK	Lake				
MOT^2^ (%)	47.3	43.8	38.7	38.2	2.4	0.184	< 0.001	0.169
Viability* (%)	64.3	59.9	53.7	50.0	2.1	0.022	< 0.001	0.770
PMOT^3^ (%)	10.0^A^	5.2^B^	3.8^B^	4.2^B^	1.0	0.003	< 0.001	< 0.001
VCL^4^ (µm/s)	36.9^a^	33.1^b^	28.0^c^	29.0^c^	1.6	0.230	< 0.001	0.014
VAP^5^ (µm/s)	22.7^a^	19.6^b^	15.6^c^	16.1^c^	1.1	0.145	< 0.001	0.012
VSL^6^ (µm/s)	15.6^A^	11.9^B^	9.2^B^	9.7^B^	0.9	0.028	< 0.001	0.001
LIN^7^ (%)	33.1	30.7	25.2	26.3	1.4	0.519	< 0.001	0.087
ALH^8^ (µm)	1.10	1.10	1.00	1.00	0.0	0.970	< 0.001	0.188
Sperm morphology								
Morphologically normal (%)	16.9	11.5	10.3	6.1	1.1	< 0.001	< 0.001	0.440
Bulb head (%)	26.4	26.1	23.4	24.2	1.1	0.783	0.005	0.614
Acrosome deformation (%)	2.5^b^	3.8^a^	3.1^ab^	2.7^b^	0.4	0.156	0.393	0.017
Midpiece deformation (%)	5.3	4.9	4.1	3.7	0.4	0.295	0.001	0.928
Bent neck (%)	5.2	5.2	4.9	5.0	0.5	0.797	0.511	0.804
Spiral tail (%)	6.2	5.6	5.9	6.3	0.7	0.863	0.792	0.165
Spermatids (%)	0.7	1.7	0.7	0.7	0.3	0.063	0.055	0.068
Other abnormalities (%)	1.0	1.0	1.2	0.8	0.2	0.128	0.767	0.314

1 SEM – standard error of the mean.

2 MOT – total motility.

3 PMOT – progressive motility.

4 VCL – curvilinear velocity.

5 VAP – average path velocity.

6 VSL straight-line velocity.

7 LIN – linearity.

8 ALH – amplitude of lateral head displacement. ^a, b, c^ between a column, values lacking a common superscript differ (*P* < 0.05). ^A, B^ between a column, values lacking a common superscript differ (*P* < 0.01).

The analysis of sperm viability showed main effects for both time (P < 0.001) and the extender type (P = 0.021), with no interaction. The EK extender maintained a higher percentage of viable cells compared to the Lake extender at both the initial evaluation (0 h) and after 24 h of storage. Nevertheless, a significant decrease in sperm viability over time was observed in both groups.

### Sperm kinematics

In contrast to %MOT, the assessment of sperm kinematics showed an Extender × Time interaction for %PMOT (P < 0.001) (Table 1). At 0 h, the EK extender showed stimulatory effect, resulting in the highest percentage of %PMOT, which was significantly higher than in the Lake extender. However, this advantage was temporary. After 24 h of storage, %PMOT in the EK group dropped, equalizing with the values recorded for the Lake extender. Interestingly, the Lake extender demonstrated a more stable kinetic profile, as the decline in %PMOT between 0 h and 24 h was not statistically significant.

Similar interaction was observed for sperm velocities, including VCL (P = 0.013), VAP (P = 0.012), and VSL (P = 0.001). Spermatozoa diluted in the EK extender achieved the highest initial velocities at 0 h. After the 24 h of storage, VCL and VAP decreased significantly in both extenders, evening the initial differences between the EK and Lake groups. Interestingly, VSL displayed a different trend. While it strongly decreased in the EK extender over time, the drop in Lake extender was much smaller. Although the VSL values in the Lake extender still decreased during the 24 h storage, this difference was only close to statistical significance (P = 0.066). This suggests that the Lake extender provides more predictable results for VSL over time.

For LIN and ALH, no interactions were detected (P > 0.05). Both parameters were exclusively affected by storage time (P < 0.001), showing a proportional decrease in LIN and ALH after 24 h, regardless of the extender used.

### Sperm morphology

The morphological evaluation showed that the level of morphologically normal spermatozoa depended on both the extender type (P < 0.001) and storage time (P < 0.001), without significant interaction (Table 1.). The EK extender provided a significantly higher percentage of normal spermatozoa than the Lake extender at all time points, although a severe time-dependent decline in normal morphology was observed in both groups after 24 h.

The analysis of specific abnormalities showed that the frequency of swollen heads and midpiece defects was influenced solely by the storage time (P < 0.01), and the extender type had no significant effect. A Extender × Time interaction was identified only for damaged acrosomes (P = 0.016). The percentage of other morphological defects, including broken necks, spiral tails, spermatids, and other deformations, remained on similar level. There were no differences related to the experimental factors (P > 0.05).

### AI outcomes

Observations of natural mating revealed that the control pairs copulated on average 1.23 ± 1.00 times per day. The overall proportion of fertilized eggs obtained from natural mating in these pairs was 69.3% ± 8.5%.

**Fig. 1:**
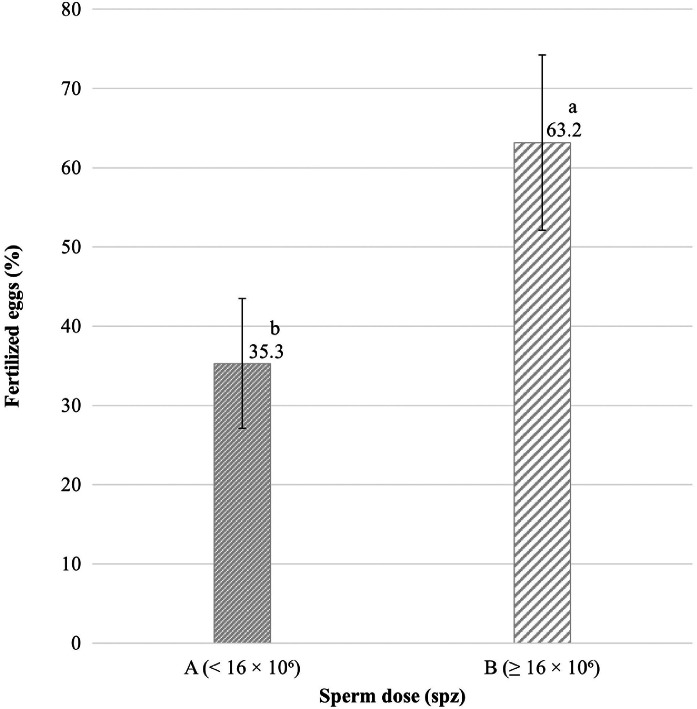
Effect of sperm dose (**A**: < 16 × 10⁶, **B**: ≥ 16 × 10⁶ live spermatozoa) on the fertilization rate of grey partridges (*Perdix perdix* L.) following artificial insemination with fresh semen. Bars represent mean ± SEM (Dose A: n = 34 eggs; Dose B: n = 19 eggs).

In the first reproductive season, when evaluating the efficacy of fresh semen (n = 23 AI events,), the GLMM analysis revealed a main effect of the insemination dose on fertilization success (P = 0.042). The application of the higher sperm dose (Dose B: ≥ 16 × 10⁶ spermatozoa) yielded a higher fertilization rate (63.2%) compared to Dose A (< 16 × 10⁶ spermatozoa; 35.3%) (Fig. 1.). The initial %PMOT of fresh individual ejaculates, included as a covariate, did not affect the fertilization outcome in this trial (P = 0.284).

**Fig. 2:**
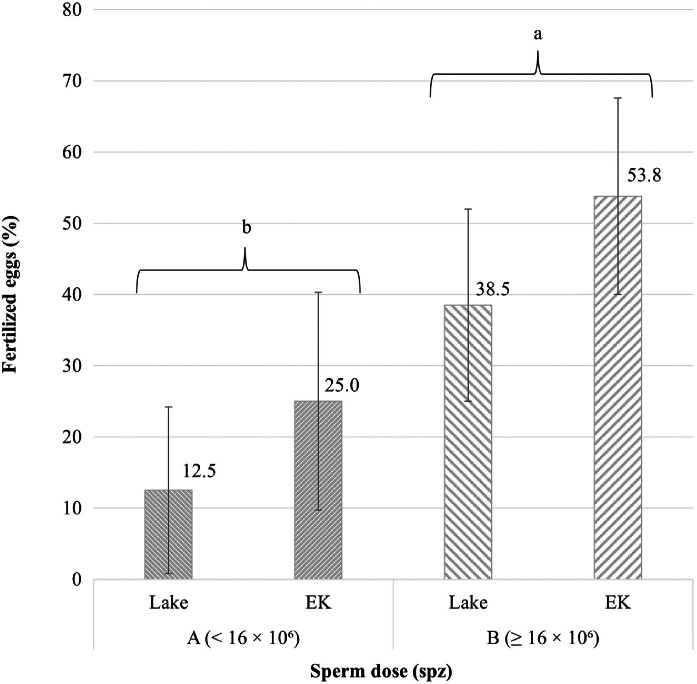
Effect of sperm dose (**A**: < 16 × 10⁶, **B**: ≥ 16 × 10⁶ live spermatozoa) and semen extender (EK vs. Lake) on the fertilization rate of grey partridges (*Perdix perdix* L.) following artificial insemination with liquid-stored semen (24 h). Bars represent mean ± SEM (Lake Dose A: n = 8 eggs; EK Dose A: n = 8; Lake Dose B: n = 13 eggs; EK Dose B: n = 13 eggs).

In the second reproductive season, when utilizing pooled semen subjected to liquid storage for 24 h (n = 24 AI events), the GLMM confirmed a main effect of the sperm dose on fertilization rates (P = 0.027). Although no significant Extender × Dose interaction was observed, dose B consistently yielded higher fertilization rates than dose A within both extender groups (EK: 53.8% vs. 25.0%; Lake: 38.5% vs. 12.5%, respectively) (Fig. 2). Regarding the type of semen extender, EK performed nominally better than Lake extender; however, this main effect did not reach statistical significance. Notably, within the liquid-stored semen trail, the initial %PMOT of the semen pool emerged as a trend influencing the fertilization rate after 24 h of storage (P = 0.06).

## Discussion

Despite the long history of AI in avian species, there is a lack of information on its application in the grey partridge (*Perdix perdix*). This study explores the feasibility of applying AI in this species and provides the first evaluation of the efficacy of short-term liquid storage of semen and subsequent in vivo fertilization. The results demonstrate that although 24-hour storage at 5 °C causes a deterioration in several semen quality parameters, AI using stored semen may serve as a valuable ex situ conservation strategy for this species in the future (Blesbois and Brillard, 2007; Blanco et al., 2009).

Studies in other avian species indicate that the first 24 h of storage constitute a critical period during which the most pronounced structural and biochemical changes in spermatozoa occur (Partyka and Niżański, 2022). In the current study, 24-hour storage of partridge semen resulted in a significant decrease in %MOT, viability, sperm velocity, and the percentage of morphologically normal spermatozoa. This pattern is consistent with observations in poultry species, where liquid storage leads to a progressive decline in semen quality due to metabolic depletion, oxidative stress, and structural damage to the sperm membrane (Donoghue and Wishart, 2000; Douard et al., 2004; Partyka and Niżański, 2021).

Whereas a general time-dependent decline in semen quality was observed, the applied extenders exhibited distinct protective profiles. The EK extender provided higher sperm viability and a greater percentage of normal morphology compared to the Lake extender, suggesting that the composition of the EK variant guarantees more favorable conditions for maintaining cellular stability during storage. This high efficacy in preserving viability and morphological integrity likely stems from EK formulation, which includes inositol, a phosphate buffer system, and a hyperosmotic pressure of 390 mOsm/kg. Inositol acts as a membrane protectant, stabilizing the sperm cell membrane against cold-induced structural damage. Recent studies on boar semen demonstrate that inositol in semen extenders possesses potent antioxidant and anti-apoptotic properties, actively decreasing the levels of oxidative stress (ROS) generated during semen storage (Sexton and Fewlass, 1978; Blanco et al., 2011; Osman et al., 2023). These findings align with observations in poultry species, where EK’s buffering capacity and nutrient composition effectively support basic cell survival (Siudzińska and Łukaszewicz, 2008; Nath et al., 2015; Rakha et al., 2016; Arıcı et al., 2025). Conversely, while EK was superior in structural preservation, our study demonstrated that the Lake extender provided a more stable kinetic profile over time. Although spermatozoa diluted in the EK extender initially showed higher %PMOT and velocity parameters, these values dropped drastically after 24 h. In the Lake extender, the decline in %PMOT and VSL was much less pronounced. Although our study focused exclusively on short-term storage, this inherent capability of the Lake extender to preserve kinetic stability is corroborated by studies on the cryopreservation of fowl semen, which showed that it can improve post-thaw %PMOT and specific kinetic parameters compared to other extenders (Salehi et al., 2020; Di Iorio et al., 2020). While the hyperosmotic and nutrient-rich conditions of EK effectively support basic cellular survival, they may simultaneously contribute to a faster draining of intracellular energy. Given that the EK extender is highly effective for prolonged liquid storage in poultry species, its different kinetic performance in the grey partridge over 24 h points to species-specific metabolic differences. It is highly plausible that partridge spermatozoa possess either lower intracellular energy reserves or higher baseline metabolic demands compared to domestic fowl. In contrast the Lake extender provides a more physiological osmotic pressure of 310 mOsm/kg, which likely prevents the rapid exhaustion of cellular ATP reserves, allowing spermatozoa to maintain their %PMOT and VSL much more consistently during the 24-hour liquid storage period (Donoghue and Wishart, 2000; Blanco et al., 2008; Siudzińska and Łukaszewicz, 2008; Słowińska et al., 2018; Arıcı et al., 2025). The specific energetic requirements of partridge spermatozoa constitute an important direction for future research.

An interesting biological finding of the present study is the remarkably low percentage of morphologically normal spermatozoa recorded in *P. perdix*. Even in fresh samples, the proportion of normal forms was significantly lower than values typically observed in poultry species. This observation aligns with our preliminary baseline investigations, which demonstrated that the proportion of morphologically normal spermatozoa in this species is only around 10% (Bawej et al., 2026). Grey partridges exhibit a strictly monogamous mating system; this high rate of abnormal forms is likely associated with a lower intensity of sperm competition. Such an evolutionary pattern is supported by broader avian studies, indicating that species characterized by low sperm competition may exhibit reduced stringency during spermatogenesis, consequently resulting in a significantly higher incidence of morphologically abnormal sperm within the ejaculate (Møller and Ninni, 1998; Sardell and DuVal, 2014; Rowe et al., 2015). Beyond this evolutionary baseline, the 24-hour liquid storage process led to a further decrease in the pool of live, morphologically intact cells. This decline, recorded irrespective of the extender type utilized, aligns with findings from other authors, confirming that such structural degradation represents a natural, time-dependent process inherent to avian semen storage (Donoghue and Wishart, 2000; Partyka and Niżański, 2022). Comparative studies on rooster semen stored in various extenders, including Lake and EK, have similarly demonstrated a universal time-dependent decline in live, normal spermatozoa. while confirming the capacity of the EK formulation to preserve normal sperm morphology across multiple breeds (Siudzińska and Łukaszewicz, 2008; Arıcı et al., 2025). The more favorable protective efficacy of the EK variant is likely associated with its higher osmolality and energy availability, which help prevent adverse morphological changes during cold storage. However, the observed numerical shifts in specific abnormalities must be interpreted in conjunction with the significant decline in overall sperm viability. The minor decrease in the percentages of certain defects, such as midpiece deformations, may be associated with the shrinking pool of live spermatozoa. As the deformed cells undergo cell death and lose membrane integrity during storage, they are classified as dead, which alters the proportion of remaining deformation categories. In contrast, the significant interaction effect for acrosome deformations suggests a more complex dynamic. It indicates that acrosomal stability is not solely dependent on storage time but is also actively modulated by the specific protective components of the extender formulation. Therefore, further research is needed to better understand the factors influencing grey partridge sperm survival in vitro and to optimize semen preservation for this species.

To establish a biological context for these findings, preliminary observations of natural mating in captive *P. perdix* pairs were recorded. The high frequency of daily copulations (average 1.23 times per day) paired with the low percentage of morphologically normal spermatozoa, indicates a specific reproductive strategy. It appears that monogamous males compensate for poor ejaculate quality through high copulation frequency, ensuring the continuous replenishment of the female's SST reserves (Birkhead et al., 1993; Bakst, 1993; Sasanami et al., 2013). This biological need for continuous sperm replenishment was clearly reflected in the in vivo trials. Following a single AI, the duration of fertility was notably short; females produced a maximum of four fertilized eggs when inseminated with fresh semen, and a maximum of three eggs when liquid-stored semen was used. After this brief window, the stored sperm reserves were depleted, and females returned to laying unfertilized eggs.

Statistical analysis confirmed that the AI dose had a clear and decisive main effect on fertilization rates. AI performed with larger semen doses (Dose B: ≥ 16 × 10⁶ spermatozoa) resulted in significantly higher reproductive success in both fresh and liquid-stored semen trials. When using liquid-stored semen, increasing the dose effectively compensated not only for the naturally low morphological quality of the partridge ejaculate but also for the deterioration in quality that occurred during the 24-hour storage period. This aligns with AI protocols developed for other poultry species, where the total number of functionally intact spermatozoa per dose is the primary determinant of success (Abouelezz et al., 2015; Mohan et al., 2018; Di Iorio et al., 2020).

The different impact of %PMOT on fertilization success between fresh and liquid stored semen provides further valuable insights into the dynamics of avian sperm transport. In the present study, initial %PMOT served as a sensitive predictor of in vivo fertility when semen was subjected to the stress of liquid storage (P = 0.06), whereas it lacked statistical significance in the fresh semen trial (P = 0.284). This difference likely results from the physiological requirements of SST colonization. As demonstrated in other studies, successful transport and SST colonization require a sufficient number of highly motile spermatozoa (Bakst, 1993; Froman, 2003). Fresh ejaculates naturally contain a high proportion of such active cells, meaning that minor individual variations in initial %MOT do not limit overall success. In contrast, 24-hour liquid storage imposes structural and metabolic stress, which significantly reduces the pool of fertilization-capable spermatozoa (Donoghue and Wishart, 2000). Under these compromised conditions, the initial %PMOT of the semen becomes a critical limiting factor. Therefore, evaluating %PMOT appears to be particularly important when assessing the fertilizing potential of stored partridge semen.

Finally, although in vitro analyses revealed distinct protective profiles for the two extenders, their in vivo performance did not differ significantly. Although the EK extender yielded a nominally higher overall fertilization rate (42.9%) compared to the Lake extender (28.6%), the GLMM analysis indicated that this numerical trend was not statistically significant (P = 0.116). However, this lack of statistical significance must be interpreted with caution due to the limited sample sizes per treatment group, which inherently reduced the statistical power to detect moderate effects. Biologically, the 14.3% nominal advantage of the EK extender is substantial and suggests a potential superiority in maintaining sperm fertilizing capacity over 24 h. This outcome underscores that avian fertility is a highly complex, multifactorial process; therefore, the biological relevance of %PMOT as a sole fertility predictor must be interpreted with caution. While progressive motility reflects the kinetic capacity of spermatozoa to reach the fertilization site, actual success depends heavily on structural functionality, plasma membrane integrity, and acrosome status, all of which dictate sperm survival and selection within the female reproductive tract (Bakst, 1993). In our study, while Lake’s kinetic stability offers technical predictability for semen handling, this effect was likely counterbalanced by EK’s higher efficacy in preserving total sperm viability and normal morphology, confirming that maintaining a sufficient pool of structurally intact, live cells is a critical driver of in vivo success. In conservation breeding programs for endangered wild species, where every fertile egg is highly valuable, such numerical trends warrant further investigation with larger cohorts when biologically feasible and imply that the EK extender remains a promising candidate for grey partridge semen preservation.

It must be emphasized that this study represents the first-ever report on the application of artificial insemination in the grey partridge. Because of the pioneering and sequential nature of this research, a direct parallel comparison between the fresh and stored semen trials was not the primary objective, and such cross-season comparisons are methodologically constrained by the absence of a parallel fresh control in the second year. However, from a biological perspective, the fertility outcomes achieved after 24 h of storage are highly encouraging. When using a sufficient sperm dose combined with the EK extender, the fertilization rate reached a satisfactory level (53.8%). This demonstrates that despite the naturally high baseline of abnormal spermatozoa and the physiological fragility of partridge semen, liquid preservation for 24 h is a viable and effective tool for conservation breeding programs.

From a practical standpoint, these results demonstrate that short-term liquid storage of grey partridge semen is feasible and can yield satisfactory fertility when an adequate dose is applied. Future research should focus on assessing the impact of longer liquid storage period (up to 48 h) and the targeted addition of supplements such as antioxidants to further stabilize the %PMOT of spermatozoa. Nevertheless, this study provides a foundation for further work on optimizing assisted reproduction, cryopreservation protocols and establishing a genetic reserve for the grey partridge.

## Declaration of artificial intelligence and ai-assisted technologies in the writing process

During the preparation of this work, the authors used Gemini (Google) for language editing, translation, and improving sentence structure, and Scopus AI (Elsevier) to support literature search and overview. These tools were used solely to improve readability and access to existing literature. The authors reviewed and edited all content and take full responsibility for the content of the publication.

## CRediT authorship contribution statement

**Marcel Bawej:** Writing – original draft, Visualization, Project administration, Methodology, Investigation, Funding acquisition, Formal analysis, Data curation, Conceptualization. **Marta Michalak:** Writing – review & editing, Visualization, Validation, Investigation, Formal analysis, Data curation. **Joanna Rosenberger:** Writing – review & editing, Validation, Supervision, Resources, Investigation, Conceptualization. **Artur Kowalczyk:** Writing – review & editing, Validation, Supervision, Resources, Conceptualization.

## Disclosures

The authors declare that they have no known competing financial interests or personal relationships that could have appeared to influence the work reported in this paper.
